# Conversion of Wheat Bran to Xylanases and Dye Adsorbent by *Streptomyces thermocarboxydus*

**DOI:** 10.3390/polym13020287

**Published:** 2021-01-17

**Authors:** Thi Ngoc Tran, Chien Thang Doan, San-Lang Wang

**Affiliations:** 1 Doctoral Program in Applied Sciences, College of Science, Tamkang University, New Taipei City 25137, Taiwan; ttngoc@ttn.edu.vn; 2 Faculty of Natural Sciences and Technology, Tay Nguyen University, Buon Ma Thuot 630000, Vietnam; dcthang@ttn.edu.vn; 3 Department of Chemistry, Tamkang University, New Taipei City 25137, Taiwan; 4 Life Science Development Center, Tamkang University, New Taipei City 25137, Taiwan

**Keywords:** agro-byproducts, dye adsorption, antioxidant, prebiotic, *Streptomyces thermocarboxydus*, xylanase, xylo-oligosaccharide

## Abstract

Agro-byproducts can be utilized as effective and low-cost nutrient sources for microbial fermentation to produce a variety of usable products. In this study, wheat bran powder (WBP) was found to be the most effective carbon source for xylanase production by *Streptomyces thermocarboxydus* TKU045. The optimal media for xylanase production was 2% (*w/v*) WBP, 1.50% (*w/v*) KNO_3_, 0.05% (*w/v*) MgSO_4_, and 0.10% (*w/v*) K_2_HPO_4_, and the optimal culture conditions were 50 mL (in a 250 mL-volume Erlenmeyer flask), initial pH 9.0, 37 °C, 125 rpm, and 48 h. Accordingly, the highest xylanase activity was 6.393 ± 0.130 U/mL, 6.9-fold higher than that from un-optimized conditions. *S. thermocarboxydus* TKU045 secreted at least four xylanases with the molecular weights of >180, 36, 29, and 27 kDa when cultured on the WBP-containing medium. The enzyme cocktail produced by *S. thermocarboxydus* TKU045 was optimally active over a broad range of temperature and pH (40–70 °C and pH 5–8, respectively) and could hydrolyze birchwood xylan to produce xylobiose as the major product. The obtained xylose oligosaccharide (XOS) were investigated for 2,2-diphenyl-1-picrylhydrazyl radical scavenging activity and the growth effect of lactic acid bacteria. Finally, the solid waste from the WBP fermentation using *S. thermocarboxydus* TKU045 revealed the high adsorption of Congo red, Red 7, and Methyl blue. Thus, *S. thermocarboxydus* TKU045 could be a potential strain to utilize wheat bran to produce xylanases for XOS preparation and dye adsorbent.

## 1. Introduction

Hemicellulose, cellulose, and lignin are the three major types of polymers that compose the plant cell-wall [1,2]. The reserve of hemicellulose is extremely abundant, only after cellulose in the plant biomass. One of the primary components of hemicellulose is xylan, a polysaccharide that comprises *β*-1,4-linked xylose units with side chains of α-glucuronic acids and α-arabinofuranose [3]. Thus, the complete depolymerization of hemicellulose may require the contribution of xylan-degrading enzymes such as endo-xylanase, exo-xylanase, feruloyl xylan esterase, α-glucuronidase, arabinase, acetyl xylan esterase, and α-L-arabinofuranosidase [4,5]. Among them, endo-xylanase, and exo-xylanase are the most important contributors to xylan depolymerization. Until now, vast applications of xylanase have been extensively explored based on its ability in xylan degradation, for example, biofuels production [6,7,8], pulp and paper bleaching [9,10,11], bioactive compounds [12,13,14], animal feed [15,16], and baking [17,18,19].

Microorganisms, such as bacteria [20,21,22,23], actinomycetes [24,25], and fungi [26,27,28,29] are the primary sources for xylanase production. Among these, xylanases are produced by various strains of *Streptomyces* [30,31,32,33,34,35,36,37,38]. However, many of those studies used commercial xylan as the carbon source for xylanase production [32,33,34,35,36]. As a result, the high price of commercial xylan may increase the cost of enzyme production and thus limit the applications of xylanase. This issue can be solved to some extent by using cheap nutrient sources as the alternative for the fermentation processes [39]. Among cheap materials, by-products are cheaper and abundant in supply, therefore, are preferred as potential sources for microbial fermentation. Accordingly, various kinds of byproducts, which contain a significant amount of proteins and carbohydrates, have been used as carbon and nitrogen sources for microbial fermentation to produce several bioactive compounds, including enzymes [40,41,42,43,44,45,46,47,48]. Some of the agro-byproducts that could give a high-efficiency in xylanase production via microbial fermentation include wheat bran, rice bran, rice straw, sugarcane bagasse, sawdust, and wheat husk [39,49]. This leads to the idea of using agro-byproducts as economically effective nutritional supplements for the synthesis of xylanase by *Streptomyces* strains.

In this study, wheat bran powder (WBP), an inexpensive agro-byproduct was utilized as the sole carbon source to produce xylanases by *S. thermocarboxydus* TKU045. This strain produced at least four xylanases on medium which contains wheat bran. Further, the conditions for xylanase production via *S. thermocarboxydus* TKU045 were explored and the enzyme cocktail obtained from those conditions was characterized. To determine the potential use, the xylan hydrolysate obtained from the hydrolysis of birchwood xylan using the enzyme cocktail was evaluated for 2,2-diphenyl-1-picrylhydrazyl (DPPH) radical scavenging activity and the effect on lactic acid bacterial growth. Finally, the dye adsorption ability was examined from the solid waste from the WBP fermentation using *S. thermocarboxydus* TKU045.

## 2. Materials and Methods

### 2.1. Materials

*S. thermocarboxydus* TKU045 was isolated and described in the previous study [50]. Wheat bran and rice bran were collected from Miaoli (Miaoli City, Taiwan). Nutrient broth (NB) and DeMan, Rogosa, and Sharpe broth (MRS) were obtained from Himedia (Mumbai, India). Birchwood xylan and xylose were purchased from Sigma Chemical Corporation (P.O. Box 14508, Saint Louis, MO, USA). Xylobiose (CAS 6860-47-5) and xylopentaose (cat. no. OXPE) were purchased from TCI, Toshima, Kita-Ku, Tokyo, Japan, and Megazyme, respectively. Xylotriose (TRC-X750405) and xylotetrose (TRC-X750410) were obtained from Toronto Research Chemicals (TRC), Canada. *Lactobacillus rhamnosus* BCRC 16000, *Bifidobacterium bifidum* BCRC 14615, *L. paracasei* subsp. *paracasei* BCRC 14023, and *L. rhamnosus* BCRC 10940, were obtained from the Bioresource Collection and Research Center (Hsinchu, Taiwan). All other chemicals were of the highest possible quality.

### 2.2. Xylanase Assay

Xylanase activity was performed by the dinitrosalicylic acid (DNS) method [51] with some modifications. In short, the mixture of the sample (50 μL) and 1% (*w/v*) birchwood xylan solution (200 μL) in phosphate buffer (50 mM, pH 7) was incubated at 37 °C for 30 min allowing depolymerization of xylan. The reaction was stopped by adding 1500 μL DNS reagent and then the mixture was heated at 100 °C for 10 min allowing the color-forming reaction. The intensity of red-brown color was measured at 515 nm on a microplate reader to estimate the concentration of reducing sugar in the reaction system. One xylanase unit was defined as the amount of enzyme needed for the liberation of one µM of reducing sugar (xylose) in one minute.

### 2.3. Agro-Byproducts as the Sole Carbon Source for Xylanase Production

A basic medium with 0.05% (*w/v*) MgSO_4_, and 0.10% (*w/v*) K_2_HPO_4_ [52] was used to investigate the optimum carbon source for xylanase production by *S. thermocarboxydus* TKU045. One percent (*w/v*) of each carbon source, including rice bran powder (RBP), wheat bran powder (WBP), spent coffee grounds (SCG), coffee husk powder (CHP), xylan from birchwood, and carboxymethyl cellulose (CMC) were added separately to the medium. The culture conditions were 37 °C, and 150 rpm and the xylanase activity of the culture medium was estimated every day. The best carbon source was then investigated for the optimum concentration for xylanase production. The effect of the concentration of carbon source was investigated in a range of 0.25–3% (*w/v*).

### 2.4. Effects of Nitrogen Sources on Xylanase Production

The medium with 2% WBP, 0.05% (*w/v*) MgSO_4_, and 0.10% (*w/v*) K_2_HPO_4_ was used to optimize the nitrogen source for xylanase production. Different nitrogenous materials such as yeast extract, peptone, beef extract, KNO_3_, and NH_4_NO_3_ were supplemented to the medium at the same concentration of 1% (*w/v*). The culture conditions were 37 °C, and 150 rpm and the xylanase activity of the culture medium was estimated every day. The best nitrogen source was then examined for the optimum concentration for xylanase production. The effect of nitrogen source concentration was investigated in a range of 0–2% (*w/v*).

### 2.5. Effects of Culture Conditions on Xylanase Production

The WBP-containing medium was used to optimize the culture condition for the xylanase production by *S. thermocarboxydus* TKU045. The medium included 2% WBP, 1% KNO_3_, 0.05% (*w/v*) MgSO_4_, and 0.10% (*w/v*) K_2_HPO_4_. The effect of the initial pH, incubation temperature and shaking speed was examined in the range of pH 7–10, 30–50 °C, and 0 rpm–175 rpm, respectively. The effect of culture medium was investigated in a range of 50–150 mL in a 250 mL flask. After every one day, 1 mL of culture medium was withdrawn to test the xylanase activity. Finally, all the optimum conditions were applied to confirm the efficiency of the optimization process.

### 2.6. Determination of Molecular Weight (MW) of the Xylanases

The MW of xylanases was determined using the sodium dodecyl sulfate (SDS)-polyacrylamide gel electrophoresis (PAGE) method [53] with some modifications. Briefly, the 10% resolving gel was co-polymerized with 0.05% birchwood xylan (*w/v*). The culture medium and crude enzyme samples were mixed with the sample buffer and then 5 µL of each sample was loaded on the prepared gel and electrophoresis was conducted at 114 V, and 4 °C. Then, residual SDS was removed by washing the gel in 2% Triton X-100 (prepared in phosphate buffer, 50 mM, pH 7), and then in phosphate buffer alone. The xylanolytic reaction was carried out by incubating the gel in phosphate buffer for 1 h at 37 °C. The staining step was conducted by dipping the gel in 0.01% (*w/v*) Congo red solution for 30 min, and then the gel was washed by 1 M NaCl solution. The xylanase activity appeared as a clear band against the red background of the gel.

### 2.7. Effects of Temperature and pH

The optimal temperature of the crude enzyme cocktail was explored by immediately incubating it with substrate solution at different temperatures (from 10 to 100 °C) for 30 min. The thermal stability of the crude enzyme cocktail solution was explored by pre-treating at different temperatures (10–100 °C) for 30 min and the residual xylanase activity was measured. The optimal pH of the crude enzyme cocktail was determined by adjusting the pH of the reaction solution. The buffer systems explored were citrate buffer (pH 3–7), phosphate buffer (pH 6–8.5), and carbonate buffer (pH 8.5–11). The pre-treated crude enzyme solution in different pH for 30 min at 4 °C, was then adjusted to pH 7 and its residual xylanase activity was measured to explore the pH stability of the crude enzyme cocktail.

### 2.8. Effect of Divalent Metal Ions, Surfactants, and Ethylenediaminetetraacetic Acid (EDTA)

Five millimolar and 10 mM concentrations of divalent metal ions (Ba^2+^, Ca^2+^, Cu^2+^, Fe^2+^, Mn^2+^, Mg^2+^, and Zn^2+^) were prepared, and the other chemicals such as EDTA, SDS, Tween 20, Tween 40, Triton X-100 and SDS were prepared at 5% and 10%. At first, an equivalent volume of the crude enzyme cocktail and each of those chemicals were mixed in a glass tube at 4 °C for 30 min and then the residual xylanase activities of the crude enzyme cocktail were then measured by xylanase assay (as mentioned above).

### 2.9. Xylan Hydrolysis

The hydrolysis solutions of 1% (*w/v*) birchwood xylan with crude enzyme cocktail were analyzed at 1:4 in ratio at 0, 0.5, 1, 2, 3, 4, 5, 6, and 22 h by thin-layer chromatography (TLC, silica gel 60, F_254_, Aluminum TLC plate, Merck). The mixture of propanol/ammonia solution/water (7/1/2, *v/v/v*) was used as the mobile phase. Finally, the TLC plate was sprayed with 10% H_2_SO_4_ in ethanol and heated at 180 °C [54]. A mixture of xylo-oligosaccharides including of xylose (X1), xylobiose (X2), xylotriose (X3), xylotetraose (X4), and xylopentose (X5) were used as the standard.

### 2.10. 2,2-Diphenyl-1-picrylhydrazyl (DPPH) Radical Scavenging Activity Assay

DPPH radical scavenging activity assay was carried out according to an earlier published report [55].

### 2.11. Growth Effect Assay

The effect of xylose oligosaccharide (XOS) was checked on the growth of *L. rhamnosus* BCRC 16000, *B. bifidum* BCRC 14615, *L. paracasei* subsp. *paracasei* BCRC 14023, and *L. rhamnosus* BCRC 10940. XOS was added to MRS medium at different concentrations (0.01%, 0.05%, and 0.10%, *w/v*). The bacterial strains were grown at 37 °C for 24 h. For *B. bifidum* BCRC 14615, 0.05% (*w/v*) cysteine was added to the medium, and the culture was carried out in anaerobic mode. To measure the relative growth cell of the bacteria on MRS culture containing XOS, A_600 nm_ of the culture MRS medium was used as the control.

### 2.12. Dye Adsorption Assay

The dye adsorption ability of solid waste obtained from the WBP fermentation by *S. thermocarboxydus* TKU045 was investigated using ten dyes, including Red 6, Yellow 4, Red 40, Red 7, Green 3, Yellow 5, Blue 1, Blue 2, Congo red, and Methyl blue. The mixture of 100 mg of adsorbent and 5 mL of dye solution (0.02%, *w/v*) was shaken for 60 min, and then the color intensity of the final solution was measured by a spectrophotometer. The adsorption rate (%) was calculated by using the formula:

Adsorption rate (%) = (A_C_ − A_S_)/A_C_

where A_C_ is the absorbance of the control solution (original dye solution), and A_S_ is the absorbance of the experimental group.

### 2.13. The Fourier-Transform Infrared Spectroscopy (FTIR) Analysis

FTIR spectra of unloaded and loaded dye samples were measured in the presence of KBr over the range of 4000–400 cm^−1^ on Nicolet iS5 FTIR spectrophotometer (Thermo, Waltham, MA, USA) [56].

### 2.14. Statistical Analysis

For the statistical analysis of the results, the data on the experiments were analyzed by Microsoft Excel 2016.

## 3. Results and Discussion

### 3.1. Agro-Byproducts as the Sole Carbon Source for the Xylanase Production

**Figure 1:**
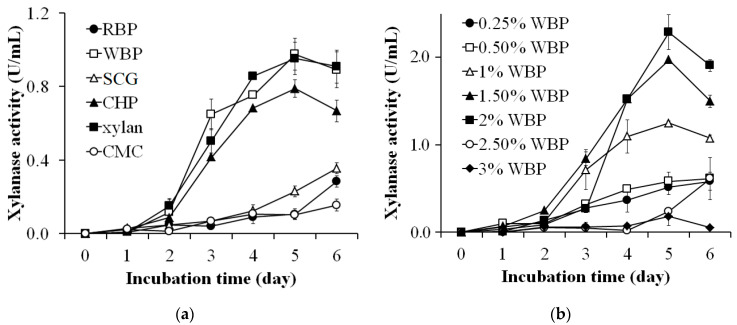
Screening of the carbon source (**a**) and amount of wheat bran powder (WBP) (**b**) for the production of xylanase by *Streptomyces thermocarboxydus* TKU045.

Various carbon sources were investigated for the preparation of an optimum culture medium for the production of xylanase by *S. thermocarboxydus* TKU045. These included RBP, WBP, SCG, CHP, birchwood xylan, and CMC. As shown in Figure 1a, the highest xylanase activity was observed in the culture supernatant using xylan or WBP as the carbon source (0.952 ± 0.088 U/mL and 0.975 ± 0.085U/mL, respectively) followed by using CHP (0.788 ± 0.047 U/mL) on the 5th day. In contrast, much lower xylanase activity was detected when CMC, RBP, and SCG were used as carbon sources. Xylan is known to be the most effective carbon source for the synthesis of xylanase by *Streptomyces* [32,33,34,35,36,38,57,58,59]. However, the high price of xylan may be a deterrent to its use in the production of xylanase. Therefore, the utilization of agro-byproducts can be an effective alternative. Accordingly, various substances have been indicated in the literature as suitable carbon sources for producing xylanase, such as rice bran [37], wheat bran [60,61,62], rice straw [33], and sugar cane bagasse [49]. In this study, WBP exhibited the highest enzyme activity among the tested byproducts and was thus chosen as the most suitable carbon source for xylanase production by *S. thermocarboxydus* TKU045.

To examine the effect of WBP concentration on the production of xylanase, the amount of WBP in the medium was adjusted in a range of 0.25–3% (*w/v*). As shown in Figure 1b, WBP at 2% was the most effective (2.288 ± 0.198 U/mL) in the production of xylanase, followed by 1.50% and 1% concentration (1.972 ± 0.003 U/mL, and 1.249 ± 0.021 U/mL respectively) on the 5th day. A lower xylanase activity was detected using WBP at other concentrations (0.25%, 0.50%, 2.50%, and 3%). Thus, the optimum carbon source used for xylanase production by *S. thermocarboxydus* TKU045 was 2% (*w/v*) WBP.

### 3.2. Effects of Nitrogen Source on Xylanase Production

**Figure 2:**
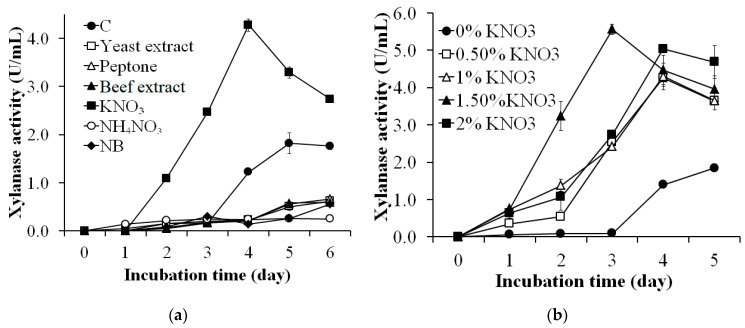
Screening of the appropriate nitrogen source (**a**) and amount of KNO_3_ (**b**) for xylanase production by *S. thermocarboxydus* TKU045. C, control medium (2% (*w/v*) WBP, 0.05% (*w/v*) MgSO_4_, and 0.10% (*w/v*) K_2_HPO_4_); NB, nutrient broth.

Different nitrogen sources such as yeast extract, peptone, beef extract, KNO_3_, and NH_4_NO_3_ were supplemented to the WBP-containing medium to assess the suitable nitrogen source for xylanase production by *S. thermocarboxydus* TKU045. Besides, NB medium supplemented with 2% WBP was also used in this investigation. Interestingly, only KNO_3_ showed a positive effect on xylanase production, whereas all other nitrogen sources had a negative impact (Figure 2a). The highest xylanase activity was observed on the 4th day using KNO_3_ as the nitrogen source (4.271 ± 0.131 U/mg). This result was 2.34-folds higher than the xylanase activity of liquid supernatant of the control group (1.820 ± 0.211 U/mL) on the 5th day. Also, the cultivation time to achieve the maximum xylanase productivity using KNO_3_ was shorter (4 days) than that of other nitrogen sources. Chi et al. (2013) reported that the best xylanase production for *S. thermocarboxydus* MW8 was observed using soytone as the nitrogen source [32]. However, Sinjaroonsak et al. (2019) found that yeast extract was the most suitable nitrogen source for xylanase production by *S. thermocoprophilus* TC13W [63].

The effect of KNO_3_ concentration on the xylanase production was studied by adding 0–2% KNO_3_ into the WBP-containing medium. As shown in Figure 2b, the maximal xylanase activity of medium with KNO_3_ at 0%, 0.50%, 1%, 1.50%, and 2% was 1.840 ± 0.013 U/mL (day 5), 4.251 ± 0.206 U/mL (day 4), 4.300 ± 0.357 U/mL (day 4), 4.476 ± 0.393 U/mL (day 4), 5.563 ± 0.137 (day 3), and 5.037 ± 0.045 U/mL (day 4), respectively. This result indicates that KNO_3_ at 1.50% concentration was the most suitable for xylanase production by *S. thermocarboxydus* TKU045.

### 3.3. Effects of Culture Conditions on Xylanase Production

**Figure 3:**
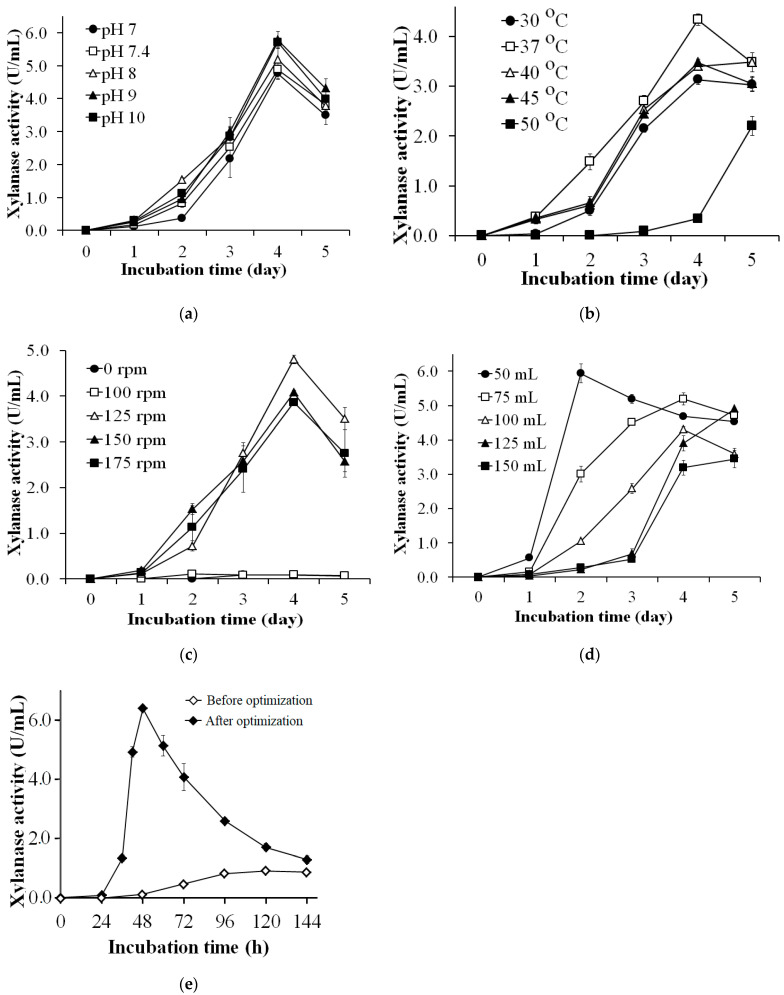
Effects of culture conditions, initial pH (**a**), temperature (**b**), shaking speed (**c**), culture medium (**d**), and incubation time (**e**) for xylanase production by *S. thermocarboxydus* TKU045.

The effect of initial pH on xylanase production was studied by adjusting the pH of WBP containing medium to pH 7, 8, 9, and 10. The medium without pH adjustment (at pH 7.4) was also studied. As shown in Figure 3a, the highest xylanase activity was observed on the 4^th^ day at 4.765 ± 0.185 U/mL (pH 7), 4.884 ± 0.290 U/mL (pH 7.4), 5.190 ± 0.326 U/mL (pH 8), 5.785 ± 0.252 U/mL (pH 9), and 5.710 ± 0.133 U/mL (pH 10). An increase in the initial pH of the medium seemed to promote more xylanase production. At pH 9 and 10, the maximum xylanase activity of medium was significantly higher than that at pH 7 and 7.4. However, the maximum enzyme activity of medium at pH 8 was not significantly different than that at other pH values. This indicated that xylanase is produced media over a wide range of pH (pH 7–10). The optimum pH of the medium for xylanase production was different from that of other *Streptomyces* strains. Some reports indicated that the optimum pH for the xylanase production was pH 8–9 using strains including *Streptomyces* sp. RCK-2010 [60], *S. cyaneus* SN32 [62], and *S. griseorubens* LH-3 [64]. In contrast, other strains like *Streptomyces sp.* B6 [35], *S. thermocarboxydus* MW8 [32], *S. olivaceoviridis* E-86 [58], and *S. rameus* L2001 could produce xylanase at optimum levels in the range of pH 6–7.2 [65].

Temperature is one of the key factors that significantly impacts microbial growth and enzyme production. In this study, we investigated a temperature range of 30–50 °C for the optimal temperature for xylanase production by *S. thermocarboxydus* TKU045. This strain gave the highest xylanase activity of 4.330 ± 0.114 U/mL at 37 °C (Figure 3b). Thus, 37 °C was selected at the most suitable temperature for the xylanase production by *S. thermocarboxydus* TKU045. Several studies have shown that an optimum temperature ≥ 40 °C for xylanase production by *S. thermocarboxydus* MW8 [32], *Streptomyces* sp. RCK-2010 [60], *S. cyaneus* SN32 [62], *S. thermovulgaris* TISTR1948 [66], and *S. variabilis* MAB3 [67]. However, some *Streptomyces* strains such as *Streptomyces* spp. SKK1-8 (30 °C) [57], and *Streptomyces* sp. P12-137 could also produce xylanase at a lower temperature (28 °C) [68].

The shaking speed of the fermentation flask plays an important role in microbial fermentation as it involves oxygen dissolution and substance transfer in the culture medium. As shown in Figure 3c, *S. thermocarboxydus* TKU045 exhibited the highest xylanase activity at the shaking speed of 125 rpm (4.803 ± 0.099 U/mL), whereas a lower activity was observed at higher or lower shaking speed. Too high shaking speed may cause higher shear stress, resulting in the decline in enzyme production while too low shaking speed may cause less homogenous mixing and low dissolved oxygen. Several studies have indicated improved production of xylanase by *Streptomyces* at a higher shaking speed (140 rpm–200 rpm) unlike that of *S. thermocarboxydus* TKU045 observed in this study [33,34,38,60,62,63,64,65].

The effect of medium volume on xylanase production was studied by adjusting the volume of WBP containing medium to 50, 75, 100, 125 and 150 mL. As shown in Figure 3d, the highest xylanase activity was observed on the 3rd day (5.934 ± 0.279 U/mL) in 50 mL medium, and less activity was observed in 75 mL, and 100 mL medium on the 4th day (5.189 ± 0.071, and 4.308 ± 0.075 U/mL, respectively), and in 125 mL and 150 mL medium on the 5th day (4.905 ± 0.0195, and 3.447 ± 0.253 U/mL, respectively). Thus, 50 mL was selected as the optimum medium volume for xylanase production by *S. thermocarboxydus* TKU045.

The optimum components of medium and culture conditions to obtain the highest xylanase productivity by *S. thermocarboxydus* TKU045 were explored. The medium components were 2% WBP, 1.50% KNO_3_, 0.05% MgSO_4_, and 0.10% K_2_HPO_4_, and the culture conditions were 50/250 mL, pH 9.0, 37 °C, and 125 rpm. To evaluate the efficiency of the optimization process, *S. thermocarboxydus* TKU045 was cultured at conditions before and after optimization. The conditions before optimization included the medium components (1% xylan, 0.05% MgSO_4_, and 0.10% K_2_HPO_4_), and culture conditions (100/250 mL, pH 8.0, 37 °C, 150 rpm). The time course of xylanase production by *S. thermocarboxydus* TKU045 in the conditions before and after optimization is shown in Figure 3e. The maximum xylanase productivity of *S. thermocarboxydus* TKU045 was 6.393 ± 0.130 U/mL (at 48th h) at the optimized conditions, and 0.932 ± 0.088 U/mL (at 120th h) in the original condition. The results indicated that *S. thermocarboxydus* TKU045 could increase the xylanase productivity in the optimized conditions by 6.9-fold than that in the original conditions. Moreover, the incubation time for maximum xylanase productivity was shortened from 5 days to about 48 h. To date, an optimal incubation time of 48 h has been reported for only a few *Streptomyces* strains for xylanase production, (for example *Streptomyces* sp. RCK-2010 [60], and *S. cyaneus* SN32 [62]), while many strains required a longer fermentation period such as *Streptomyces* sp. SWU10 (3 days) [33], *S. thermocarboxydus* MW8 (4 days) [32], *S. matensis* DW67 (5 days) [34], *S. chartreusis* L1105 (7 days) [38], and *Streptomyces* spp. SKK1-8 (10 days) [57]. The longer fermentation period may increase the production cost, therefore, a shorter fermentation is more advantageous. Besides, wheat bran is a cheap and largely available agro-byproduct, suggesting that it is a suitable carbon source for microbial fermentation processes on a larger scale. Taken together, the conversion of wheat bran by *S. thermocarboxydus* TKU045 could be considered as a cheap and efficient way in the xylanase production process.

### 3.4. Molecular Weight Determination of the Xylanases

**Figure 4:**
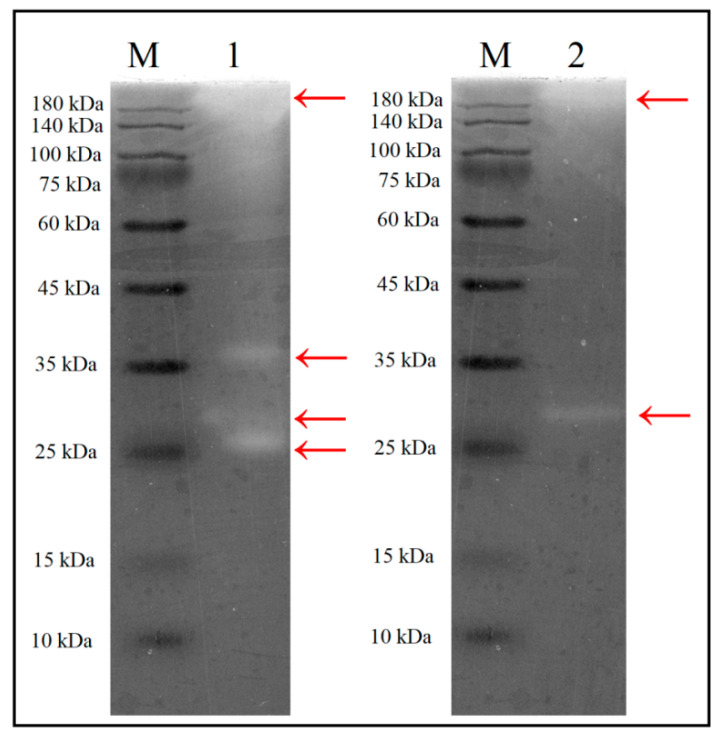
SDS-PAGE zymography of the culture supernatant and crude enzyme after (NH4)_2_SO_4_ precipitation step. 1, Culture supernatant; 2, crude enzyme; M, protein markers. *S. thermocarboxydus* TKU045 was cultured in the optimized medium at optimal conditions. Then, the culture medium was centrifuged at 13,000 rpm to collect the culture supernatant.

To determine the expression pattern of the xylanases, the secretome of *S. thermocarboxydus* TKU045 over WBP-containing medium was analyzed by SDS-PAGE containing 0.05% xylan. As shown in Figure 4, at least four different xylanases with >180, 36, 29, and 27 kDa of MW were observed indicating that TKU045 produced multiple xylanases into the culture medium to effectively degrade the complex substance such as wheat bran. Interestingly, (NH_4_)_2_SO_4_, a common chemical for enzyme concentration, could only precipitate the 29 and >180 kDa xylanases. The SDS-PAGE zymography result of the crude enzyme in Figure 4 reveal only >180 and 29 kDa bands of xylanase activity, whereas 36 and 27 kDa bands not present. The production of multiple xylanases has also been observed in several *Streptomyces* strains for example *Streptomyces* sp. SWU10, *Streptomyces* sp. B-12-2, *S. actuosus* A-151, *Streptomyces* sp. P12-137, and *S. olivaceoviridis* E-86 (Table 1). In general, the MW of xylanase from *Streptomyces* strains is in the range of 15–50 kDa. Thus, the >180 kDa xylanase produced by TKU045 in this study may be an exception. Furthermore, the MW of TKU045 xylanases was remarkably different from that obtained from *S. thermocarboxydus* HY-5 (43.962 Da) and *S. thermocarboxydus* MW8 (52 kDa). Therefore, these results might be considered as a novel observation towards xylanase production from *Streptomyces*.

### 3.5. Biochemical Characterization of Crude Enzyme Cocktail

**Figure 5:**
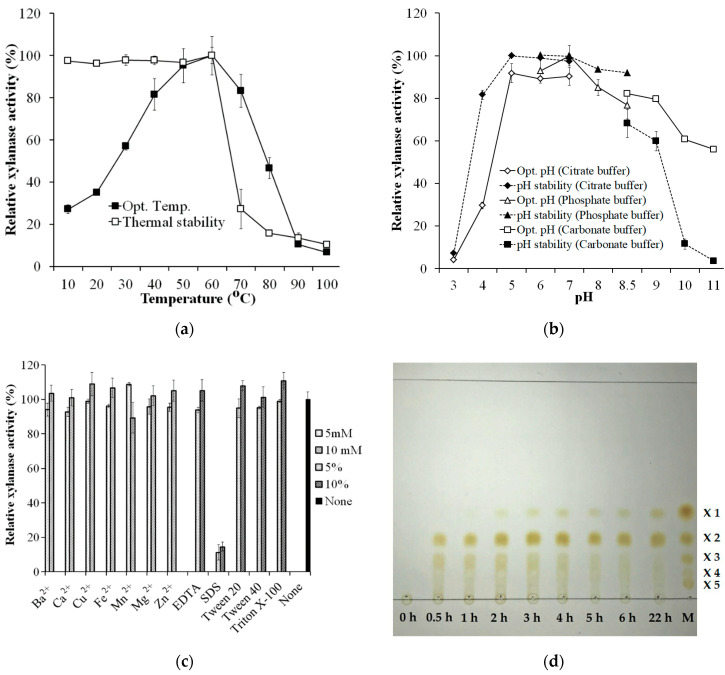
Effect of temperature (**a**), pH (**b**), and various chemicals (**c**) on the activity of *S. thermocarboxydus* TKU045′s crude enzyme cocktail; and TLC profile of birchwood xylan hydrolysis catalyzed by the enzyme cocktail (**d**).

The optimum temperature and pH of *S. thermocarboxydus* TKU045′s crude enzyme cocktail were determined using birchwood xylan as the substrate. The optimum temperature range of the crude enzyme was determined to be between 40–70 °C (Figure 5a). At temperatures beyond this range, the activity of the crude enzyme was significantly lower than that of optimum temperatures. The crude enzyme was thermally stable up to 60 °C, indicating that *S. thermocarboxydus* TKU045′s crude enzyme cocktail had high thermo-activity and thermo-stability. Thermo-activity and -stability are the common features of xylanases from *Streptomyces*. Liu et al. (2020) reported that two xylanases from *Streptomyces* sp. B6, XynST10 and XynST11, have a high thermo-activity (60 °C, and 60–90 °C, respectively) and thermo-stability (50 and 60 °C, respectively) [35]. The highest xylanase activity of *S. thermocarboxydus* TKU045′s crude enzyme cocktail was measured at pH 7 (in phosphate buffer). However, more than 80% of the enzyme activity was also observed at pH 5, 6, and 8, indicating the activity of enzyme cocktail over a broad optimum pH from pH 5–pH 8. Likewise, the enzyme cocktail was the most stable in the range of pH 5–pH 8 (Figure 5b). The broad optimal pH and pH stability may provide the enzyme a greater ability to act at various pH conditions, as was observed in the enzyme cocktail from *S. thermocarboxydus* TKU045.

The effect of metal ions on the activity of *S. thermocarboxydus* TKU045′s crude enzyme cocktail was also studied by adding the respective metal salts in the reaction mixture as described in the method section. As shown in Figure 5c, there was no significant effect of all the tested ion metals (Ba^2+^, Ca^2+^, Cu^2+^, Fe^2+^, Mn^2+^, Mg^2+^, and Zn^2+^) on the xylanase activity of crude enzyme cocktail at 5 mM and 10 mM concentrations. In contrast, Ca^2+^, Cu^2+^, Fe^2+^, Mn^2+^, Mg^2+^, and Zn^2+^ were reported to inhibit *S. thermocarboxydus* MW8 xylanase [32]. Likewise, Kim et al. (2010) reported that Cu^2+^ strongly inhibited *S. thermocarboxydus* HY-5 xylanase [31]. EDTA showed no apparent effects on the activity of *S. thermocarboxydus* TKU045′s crude enzyme cocktail, indicating that the xylanases do not need metal ions as the cofactor. Nonionic surfactants (Tween 20, Tween 40, and Triton X-100) did not show any significant effect on the xylanase activity of crude enzyme cocktail at 5% and 10% concentrations. However, SDS, an anionic surfactant strongly inhibited the enzyme activity.

The hydrolysis of xylan by *S. thermocarboxydus* TKU045 xylanases was analyzed by the TLC method. As shown in Figure 5d, the products of the process could be observed after 30 min of the initiation of hydrolysis, which revealed the highest intensity of the band of xylose dimer, followed by the band of xylose trimer. This indicates that the enzymes rapidly degrade xylan to release xylobiose and xylotriose. The occurrence of xylose oligomers with DP ≥ 3 was attributed to endo-type xylanase, whereas the occurrence of xylobiose was attributed to exo-type xylanase. Over time, the intensity of spots of xylose oligomers with DP ≥ 3 decreased and the spot of xylose appeared from 1 h of the initiation of hydrolysis. These results indicate that the cocktail of *S. thermocarboxydus* TKU045 xylanases has the exo-type activity, which could degrade xylose oligosaccharides with DP ≥ 3 to xylose and xylobiose [5]. Besides, the intensity of xylobiose did not deteriorate during the reaction period, suggesting that the exo-type enzyme(s) of TKU045 xylanases could not act on xylobiose to release xylose monomer. In short, a mixture of xylose oligosaccharides, with DP = 2 as the main component, could be obtained from the xylan hydrolysis catalyzed by the cocktail of *S. thermocarboxydus* TKU045 xylanases.

### 3.6. Antioxidant Activity and the Growth Effect on Lactic Acid Bacteria of Xylan Hydrolysate

**Figure 6:**
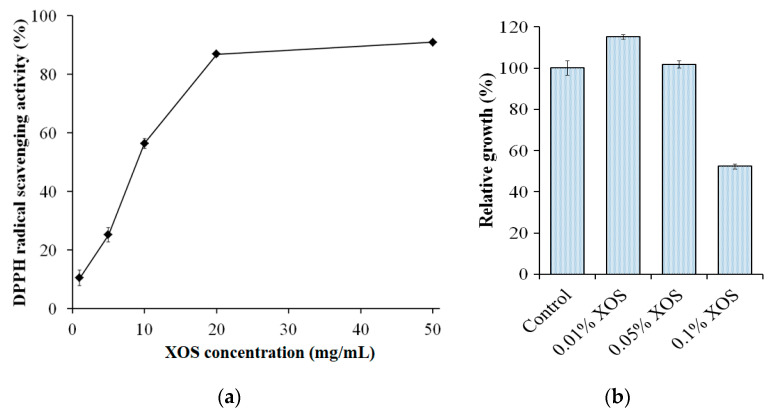
2,2-diphenyl-1-picrylhydrazyl (DPPH) radical activity (**a**) and growth effect on *Bifidobacterium bifidum* BCRC 14615 (**b**) of xylo-oligosaccharide obtained from the hydrolysis of birchwood xylan with *S. thermocarboxydus* TKU045’s crude enzyme cocktail.

Natural materials have recently been shown to be a very promising source of antioxidants, for example, peptides [53], chitosan-oligosaccharides [70], polyphenols [71], flavonoids [72], and others [71,72]. In the search for a potential antioxidant, XOS was found to possess a good free radical scavenging capacity and thus could be considered as a potential source [73]. In this study, the XOS obtained from the hydrolysis of birchwood xylan with *S. thermocarboxydus* TKU045’s crude enzyme cocktail was studied by the DPPH radical scavenging activity. As shown in Figure 6a, the DPPH radical activity of the XOS was dose-dependent at 1 mg/mL–20 mg/mL of XOS and the maximum scavenging activity was 87–91% at 20 mg/mL–50 mg/mL XOS. Huang et al. (2019) reported that XOS from Moso bamboo prehydrolyzate could achieve 86% DPPH radical scavenging activity [74]. Likewise, the birchwood xylan XOS prepared by *Bacillus amyloliquifaciens* NRRL B-14393 xylanase exhibited the maximum DPPH radical scavenging activity of 87% [75]. However, the DPPH radical scavenging activity of corncob XOSs prepared by *B. aerophilus* KGJ2 xylanase could only reach the maximum value of 74.2% [73].

XOSs have been described as a potential prebiotic and are gradually being widely recognized. However, in several reports, XOS also show antimicrobial activity. In this study, the growth effect of XOS from the hydrolysis of birchwood xylan with *S. thermocarboxydus* TKU045’s crude enzyme cocktail was examined on four lactic acid bacterial strains, including *B. bifidum* BCRC 14615, *L. rhamnosus* BCRC 16000, *L. rhamnosus* BCRC 10940, and *L. paracasei* subsp. *paracasei* BCRC 14023 at the concentration of 0.01–0.10% of XOS. Notably, 0.10% XOS exhibited a growth-inhibiting effect (52%), and 0.01% XOS had a growth-enhancing effect (115%) on *B. bifidum* BCRC 14615 (Figure 6b), indicating that the growth effect of the XOS may depend on its concentration. In contrast to the prebiotic activity, the antibacterial activity of XOS has been rarely reported. Christakopoulos et al. (2002) reported that acidic XOSs produced from birchwood xylan showed antimicrobial activity against *Staphylococcus aureus*, *B. cereus, Micrococcus flavus, Escherichia coli,* and *Helicobacter pylori* [76]. Likewise, Kallel et al. (2014) reported that XOS from garlic straw-extracted xylan had high antimicrobial activity against *Pseudomonas aeruginosa*, *B. thuringiensis*, *Enterococcus faecalis*, and *Klebsiella pneumoniae* [77]. In contrast, in the concentration range of 0.01–0.10%, the XOS did not show a significant effect on the growth of *L. rhamnosus* BCRC 16000, *L. rhamnosus* BCRC 10940, and *L. paracasei* subsp. *paracasei* BCRC 14023 (data not shown). This indicates that the XOS obtained from the hydrolysis of birchwood xylan with *S. thermocarboxydus* TKU045’s crude enzyme cocktail showed the selective growth-enhancing effect on a limited number of microbes such as *Bifidobacterium*. Similar results of the selectively growth-enhancing effect of XOSs were reported in other studies [78,79,80].

### 3.7. Utilization of Solid Waste from the WBP Fermentation as a Dye Adsorbent

**Figure 7:**
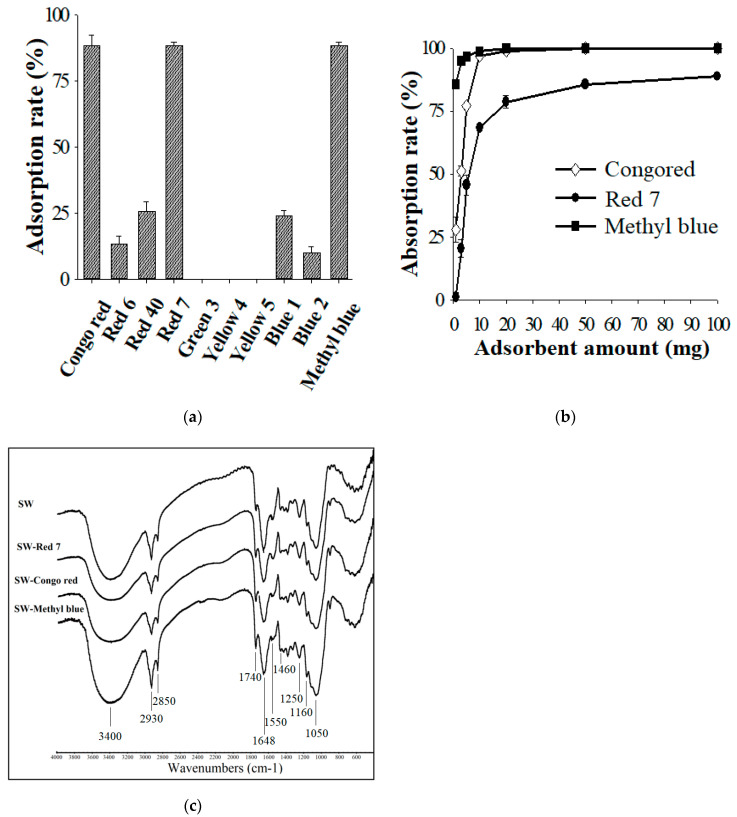
Dye adsorption ability of solid waste (SW) on Red 6, Yellow 4, Red 40, Red 7, Green 3, Yellow 5, Blue 1, and Blue 2, Congo red, and Methyl blue (**a**); Effect of the SW amount on the adsorption of Congo red, Red 7, and Methyl blue (**b**); Fourier-transform infrared spectroscopy (FTIR) profiles of SW before and after adsorbing Congo red, Red 7, and Methyl blue (**c**).

Solid waste (SW) obtained from the WBP fermentation contained residual WBP and biomass of *S. thermocarboxydus* TKU045. The high dye adsorption efficiency of wheat bran and *Streptomyces* biomass [81,82,83,84] inspired us to utilize the solid waste from the WBP fermentation as a dye adsorbent. As shown in Figure 7a, SW showed the highest adsorption rate of Congo red (89 ± 4%), Red 7 (88 ± 1%), and Methyl blue (88 ± 1%), whereas < 30% of adsorption rate was observed for Red 6, Red 40, Green 3, Yellow 4, Yellow 5, Blue 1, and Blue 2. Thus, SW holds great potential in removing Congo red, Red 7, and Methyl blue from wastewater. As shown in Figure 7b, the maximum Congo red, Red 7, and Methyl blue adsorption rates of SW were calculated to be 100%, 89%, and 100%, respectively. Further by adding 200 mg of the adsorbent, the Red 7 adsorption rate of SW reached 94% (data not shown). The FTIR spectrum profile reveals several peaks indicating that SW is composed of numerous functional groups that potentially support its binding to the dye (Figure 7c). SW exhibited a broad and strong absorption peak at 3400 cm^−1^, corresponding to the associated hydroxyl and phenolic hydroxyl groups in the cellulose. The peaks at 2930 and 2850 cm^−1^ were attributed to C-H vibrations. The peak at 1740 cm^−1^ was a carbonyl stretching vibration peak, probably due to the presence of hemicellulose, or lignin. The peak at 1648 cm^−1^ was attributed to -NH stretching, whereas, the peak at 1550 cm^−1^ indicated C–N stretching. The peak at 1460 cm^−1^ indicated the presence of C–H bending, and the peak at 1250 cm^−1^ was due to the C–O group. The broad peaks at 1160 cm^−1^ and 1050 cm^−1^ were assigned to C–O stretching from C–O–H and C–O–C bonds. The FTIR spectrum profiles of dye-loaded SWs showed a similar pattern to that of the original SW except for some slight changes in the intensity, suggesting that the adsorption mechanism of SW belonged to physical adsorption. As such, this finding provides a potential use of the waste from WBP fermentation as a low-cost and efficient sorbent.

## 4. Conclusions

In this study, a medium containing WBP as the sole carbon source was optimized for xylanase production by *S. thermocarboxydus* TKU045. From the WBP-containing medium, four xylanases of MW > 180, 36, 29 and 27 kDa were explored. The enzyme cocktail obtained by growing *S. thermocarboxydus* TKU045 on the optimal conditions was characterized. The enzyme cocktail generated xylobiose as the primary product. The xylan hydrolysate displayed DPPH radical scavenging activity and affected the growth of *B. bifidum* BCRC 14615. Finally, the solid waste from the WBP fermentation revealed the dye adsorption ability on Congo red, Red 7, and Methyl blue. Therefore, WBP fermentation by *S. thermocarboxydus* TKU045 may have potential applications in food, medicinal, and wastewater treatment. Furthermore, purification and characterization of xylanolytic enzymes from *S. thermocarboxudus* TKU045 will be further investigated.

## Figures and Tables

**Table 1:** Production of xylanase by different *Streptomyces* strains.

Strain name	Carbon and Nitrogen Sources	Culture Conditions	MW of Xylanase(s)	Ref.
*S. thermocarboxydus* TKU045	2% WBP, and 1.50% KNO_3_	50/250 mL, pH 9.0, 37 °C, 125 rpm, 48 h	>180, 36, 29, and 27 kDa	this study
*S. thermocarboxydus* HY-5			43.962 Da	[31]
*S. thermocarboxydus* MW8	1% soytone, 0.50% birchwood xylan	pH 7.0, 40 °C, 4 d	52 kDa	[32]
*Streptomyces* sp. SWU10	1% ground rice straw, and 0.20% NaNO_3_	1/3 L, 37 °C, 200 rpm, 3 d	31 and 44 kDa	[33]
*S. matensis* DW67	1.50% corncob xylan, 0.40% yeast extract, and 0.80% tryptone	50/250 mL, 30 °C, 150 rpm, and 5 d	21.2 kDa	[34]
*Streptomyces* sp. B6	0.20% beechwood xylan, and 0.02% peptone	pH 7.2, 5–7 d	48 and 33 kDa	[35]
*Streptomyces* sp. B-12-2	1% oat spelt xylan		40.5, 36.2, 36.2, 26.4, and 23.8 kDa	[36]
*S. actuosus* A-151	5% rice bran		45, 30, 26, and 20 kDa	[37]
*S. chartreusis* L1105	2.50% corncob xylan, 0.50% yeast extract, and 1% tryptone	50/250 mL, pH 6, 40 °C, 140 rpm, and 7 d	34.2 kDa	[38]
*Streptomyces* sp. RCK-2010	2.50% wheat bran, 1.20% (N_2_ equivalent) beef extract, and 0.20% (N_2_ equivalent) peptone	pH 8.0, 40 °C, 200 rpm, 48 h		[60]
*Streptomyces* sp. CS428	wheat bran		37 kDa	[61]
*Streptomyces* spp. SKK1-8	1% yeast extract, 10.30% sucrose, and 0.50% birchwood Xylan	30 °C, 140 rpm, and 10 d	15.21, 16.8, and 13.8 kDa	[57]
*S. olivaceoviridis* E-86	1.50% corncob xylan, and 1.50% Tryptone	100/500 mL, pH 6.0, 30 °C, 140 rpm, and 5 d	23 and 47 kDa	[58]
*S. cyaneus* SN32	3% wheat bran, and 1% peptone	250 mL flask, 50 mL, pH 9.0, 42 °C, 200 rpm, 48 h	20.5 kDa	[62]
*Streptomyces* sp. S38	1% oat-spelleds xylan		24.5, 37.5, and 38 kDa	[59]
*Streptomyces* sp. P12-137	1% wheat bran, 1% KNO_3_, and 0.50% xylose	50/250 mL, pH 7.2, 28 °C, and 120 h		[68]
*S. thermocoprophilus* TC13W	1% pretreated oil palm empty fruit bunch, and 0.50% yeast extract	pH 6.5, 150 rpm, 40 °C, and 120 h		[63]
*S. variabilis* MAB3	2% birchwood xylan	pH 8.2, 46.5 °C, and 68 h	50 kDa	[67]
*S. thermovulgaris* TISTR1948	2.70% rice straw, and 0.56% yeast extract	50/250 mL, pH 7.09, 50.01 °C, and 4 d	46.2 kDa	[66,69]
*S. rameus* L2001	2.50% corncob xylan, and 0.50% yeast extract	50/250 mL, 140 rpm, 40 °C, pH 6, 7 d	21.1 kDa	[65]
*S. griseorubens* LH-3	3% bagasse semi-cellulose, and 3% yeast extract	50/250 mL, pH 8, 37 °C, 160 rpm, and 96 h	45.5 kDa	[64]

## Data Availability

The data presented in this study are available on request from the corresponding author.
